# Regulating telomere length from the inside out: the replication fork model

**DOI:** 10.1101/gad.280578.116

**Published:** 2016-07-01

**Authors:** Carol W. Greider

**Affiliations:** Department of Molecular Biology and Genetics, Johns Hopkins University School of Medicine, Baltimore, Maryland 21205, USA;; Department of Biology, Johns Hopkins University, Baltimore, Maryland 21218, USA

**Keywords:** DNA replication, telomerase, telomere

## Abstract

Telomere length is regulated around an equilibrium set point. Telomeres shorten during replication and are lengthened by telomerase. Disruption of the length equilibrium leads to disease; thus, it is important to understand the mechanisms that regulate length at the molecular level. The prevailing protein-counting model for regulating telomerase access to elongate the telomere does not explain accumulating evidence of a role of DNA replication in telomere length regulation. Here I present an alternative model: the replication fork model that can explain how passage of a replication fork and regulation of origin firing affect telomere length.

## Abstract (precis)

In this Hypothesis, Greider describes a new model for telomere length regulation, which links DNA replication and telomere elongation.

Telomere length homeostasis is essential for cell survival. Short telomeres trigger DNA damage, induce cellular senescence and apoptosis, and cause short telomere syndromes and associated age-related disease (Armanios 2009). Cancer cells, on the other hand, maintain or elongate telomeres and escape senescence to allow immortal growth (Greider 1999). Telomeres naturally shorten during DNA replication, which is counterbalanced by de novo addition of telomere sequences by telomerase (Greider and Blackburn 1985). Most of the telomere is replicated by conventional replication machinery (Wellinger and Zakian 2012); however, at each cell cycle, telomerase elongates a few telomeres by addition of a few repeats (Teixeira et al. 2004). The central question is: What determines whether a telomere will be elongated and how does this establish length homeostasis? Here I present a model for how the stochastic elongation of telomeres at each cell cycle can be explained by coupling between DNA replication and telomere length maintenance.

## Telomere-binding proteins regulate telomere length

Telomeres are made up of simple G-rich DNA sequence repeats that are packaged into chromatin (Tommerup et al. 1994) and bound by telomere-specific binding proteins. In mammalian cells, the shelterin complex consists of TRF1 and TRF2, which bind along the double-stranded telomere sequence and recruit associated proteins TIN2, TPP1, POT1, and RAP1 (Palm and de Lange 2008). POT1 binds tightly to the single-stranded G-rich telomere DNA sequence. Telomeres in *Saccharomyces cerevisiae* were initially reported to be nonnucleosomal (Wright et al. 1992); however, recent data suggest nucleosomal packaging in yeast as well as mammalian cells (Rossetti et al. 2001; Pisano et al. 2008). In *S. cerevisiae*, the Rap1 protein binds to the double-stranded telomere repeats and either Rif1 and Rif2 or Sir3 and Sir4 bind to the C-terminal domain of Rap1 (Shore and Bianchi 2009). The single-stranded G-rich telomeric DNA is bound by Cdc13 (Lin and Zakian 1996; Nugent et al. 1996) and the associated Stn1 and Ten1 proteins (Grandin et al. 1997, 2001). The double-stranded and single-stranded telomere-specific binding proteins are essential for both protecting the chromosome end and regulating telomerase access to the telomere (Palm and de Lange 2008; Wellinger and Zakian 2012). How they carry out these functions is critical to understanding length regulation.

## Protein-counting model

**Figure 1.**
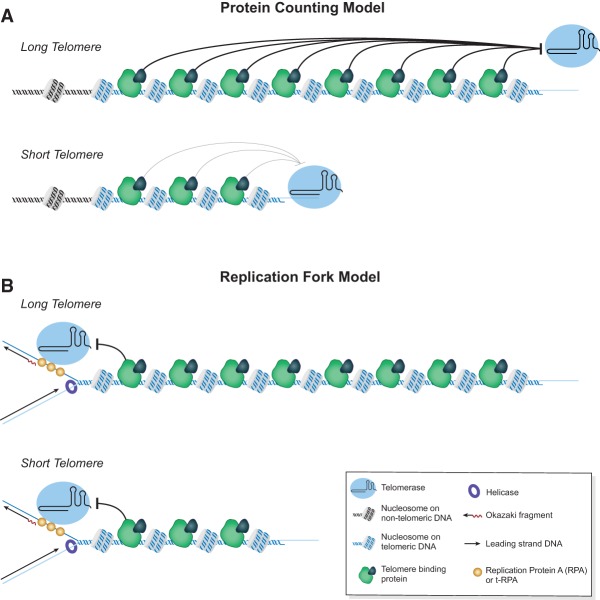
Old and new models for regulating elongation of telomeres by telomerase. (*A*) Protein-counting model: Telomeric DNA (blue helix) is shown packaged as nucleosomes and bound by interspersed telomere-specific proteins (green). The telomere proteins act from a distance to block telomerase (light blue) access to the end of the chromosome. (*Top*) The long telomere has greater repressive effects (black bar) on telomerase than the short telomere. (*B*) Replication fork model: Telomerase is shown traveling with the lagging strand machinery. The RPA or t-RPA is shown in gold, and the helicase is shown as a purple ring. The fork replicates through nucleosomes and bound telomere proteins, either of which can cause dissociation of telomerase from the fork (curved blocking bar). Telomerase must remain bound to the fork until it reaches the extreme terminus for the telomere to be extended.

Several experimental findings helped establish the “protein-counting” model for telomere length regulation (Marcand et al. 1997). First, in *S. cerevisiae*, both C-terminal mutations in *RAP1* (Sussel and Shore 1991) and deletion of the genes encoding two Rap1-interacting proteins, *RIF1* and *RIF2*, cause excessive telomere elongation (Hardy et al. 1992; Wotton and Shore 1997), implying that these proteins normally block telomere elongation. Second, addition of extra artificial RAP1-binding sites shortens telomeres (Marcand et al. 1997). Third, short telomeres are more likely to be elongated by telomerase than long telomeres (Marcand et al. 1999; Teixeira et al. 2004). The “protein-counting” model for telomere length regulation (Fig. 1A) was first described to explain length regulation in yeast (Marcand et al. 1997) and was later adopted to explain mammalian telomere length regulation, since knockdown of the telomere-binding proteins TRF1, TRF2, POT1, and TIN2 also caused excessive telomere elongation (van Steensel and de Lange 1997; Loayza and de Lange 2003; Ye and de Lange 2004; Takai et al. 2010). The evolutionary conservation of negative telomere length regulation by telomere-binding proteins helped solidify the protein-counting model (Smogorzewska et al. 2000).

At its core, the protein-counting model states that there is an additive negative effect of telomere-bound proteins on telomerase access to the telomere. That is, long telomeres have a stronger repressive effect that keeps telomerase off the 3′ end of the telomere, while short telomeres have a weaker repressive effect and so telomerase can elongate them (Fig. 1A). Although this model explains the negative inhibitory role of telomere-binding proteins, it is unclear from a biophysical standpoint how an additive negative effect might be integrated and/or propagated along many kilobases of telomere sequence. Several modifications to this model have been proposed that involve looping of the telomere DNA. Grunstein (1997) proposed that long-distance interactions of telomere-bound proteins and subtelomeric nucleosomes sequester the telomere terminus. de Lange and colleagues (Griffith et al. 1999) proposed that t loops in which the 3′ telomere end is base-paired with internal telomere repeats can regulate access of telomerase to the telomere. Finally, Lingner and colleagues (Teixeira et al. 2004) proposed a probabilistic model in which long telomeres are in a nonextendable state that can switch to an extendable state, and short telomeres have a higher probability of switching. While these models propose ways to potentially block telomeres from elongation, the mechanisms by which looping or “transitions in state” establish the exquisite normal distribution of telomere lengths are not clear.

The protein-counting model might suggest that telomere-binding proteins evolved primarily to regulate telomere length; however, even in the absence of telomerase, telomere-binding proteins regulate cell viability (Hockemeyer et al. 2008; Chang and Rothstein 2011; Ballew and Lundblad 2013). Finally, the protein-counting model does not explain a number of new experimental findings, as discussed below, suggesting that alternative models should be considered.

## A replication fork model for telomere length regulation

An alternative model for telomere length regulation better accounts for new (and old) research linking DNA replication and telomere elongation. This “replication fork” model accounts for both negative regulation of telomere elongation and preferential elongation of short telomeres. In this model, telomerase travels with the replication fork and must be deposited at the end of the telomere for that telomere to be elongated (Fig. 1B). Telomere-binding proteins (and perhaps nucleosomes) exert a negative effect by increasing the probability that telomerase will dissociate from the traveling replication fork. Therefore, the longer the telomere, the lower the probability of telomerase reaching the end, where it can preform its catalytic function. On a longer telomere, the cumulative small probabilities of telomerase dissociation make it less likely that telomerase will arrive at the terminus. This model fits the long-established evidence that short telomeres are preferentially elongated and that telomere elongation is stochastic; only a few telomeres are elongated at every cell cycle. This model also explains how telomere-binding proteins negatively regulate telomere length: They may provide a simple barrier, like the nucleosome, or some may actively promote dissociation of telomerase from the fork.

There are precedents for proteins traveling with the replication fork, including Mrc1 and Tof1, which form the fork progression complex (Katou et al. 2003). DDK travels with the replication fork to regulate double-strand breaks in meiosis (Murakami and Keeney 2014), and RRM3 travels with the fork to promote replication through specific barriers (Azvolinsky et al. 2006). The FACT complex, involved in chromatin remodeling, and Dia2, involved in replication termination, are also tethered to the replisome (Morohashi et al. 2009; Foltman et al. 2013).

There is early evidence from ciliates that telomerase also travels with the replication fork. In hypotrichous ciliates, replication initiation and progression are coordinated across the macronucleus in a “replication band” (Olins et al. 1989). This band progresses synchronously across the nucleus synthesizing DNA. The Cech laboratory (Fang and Cech 1995) showed that telomerase associates with these replication bands in *Oxytricha* as it travels with the replication forks during S phase. The coordination of replication fork progression and telomerase delivery to the very end would help explain why telomerase elongates telomeres only at the very end of S phase.

The relative stoichiometries of telomerase and replication forks may explain the stochastic nature of telomere elongation. The concentration of telomerase in vivo is very low; in *S. cerevisiae*, there are ∼20 copies of telomerase per cell, and in human cancer cell lines, there are –250 copies of telomerase per cell (Mozdy and Cech 2006; Xi and Cech 2014). In *S. cerevisiae*, there are ∼626 unique origins as well as >200 rDNA origins, a subset of which fires each cell cycle (Siow et al. 2012); thus, telomerase might associate with only a small fraction of the forks as they travel to the ends of chromosomes. As discussed below, telomerase may also have a higher probability of associating with telomeric forks, since it binds to an alternative, telomere-specific RPA.

## Origin placement and firing efficiency could regulate telomere length

**Figure 2.**
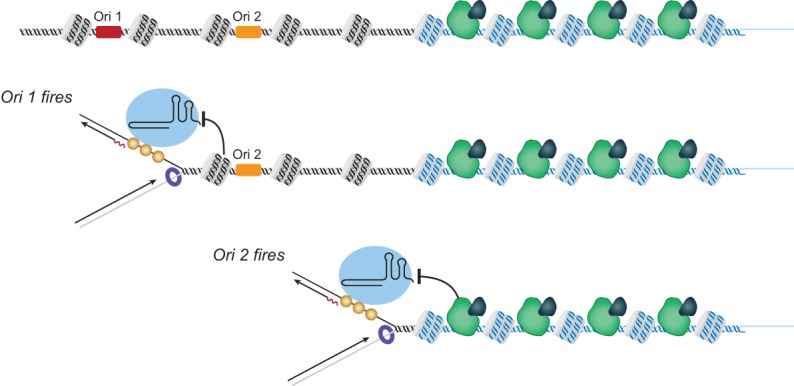
Distance from an origin may affect telomere length. Telomere-proximal origins are inhibited from firing and can be passively replicated by adjacent origins. Here, Ori 1 is efficient, while Ori 2 does not fire in every cell cycle. If telomerase travels with the fork that initiates at Ori 1, the probability of it reaching the end is relatively low. In contrast, if Ori 2 fires, there is a shorter distance to the chromosome end, and telomerase has a high probability of elongating that telomere. Rif1 normally blocks the telomeric Ori 2 from firing; in the absence of Rif1, Ori 2 will fire, and telomeres will elongate.

The replication fork model provides a plausible explanation of two previously mystifying results: how subtelomeric sequences and the regulation of origin firing both affect telomere length. Both origin location and nonfiring of a telomeric origin will affect how far a fork must travel before it reaches the chromosome end (Fig. 2). The probability that telomerase will remain bound to the replication fork until it reaches the end of the chromosome will increase with a shorter distance between the most telomere-proximal origin and the chromosome end.

The differential locations of origins may explain a curious discovery in the Petes laboratory (Craven and Petes 1999) that the lengths of telomeres containing a Y′ subtelomeric sequence are regulated differently than telomeres containing a subtelomeric X sequence. Telomeres in *S. cerevisiae* contain two types of repetitive subtelomeric sequences (termed X or Y′) immediately adjacent to the G-rich telomere repeats (Chan and Tye 1983). Both of these elements contain replication origins, but the distance of the origin from the chromosome end varies in the two repeats (Louis 1995). The replication fork model of telomere length regulation would suggest that differential proximity to an origin in X- and Y′-containing telomeres could result in different probabilities of telomere elongation (Fig. 2).

## Replication origin firing regulates telomere length

A second curious finding that can be explained by the replication fork model is the role of Rif1 in regulating origin firing and telomere length. Telomeric origins replicate late in S phase, often do not fire, and are passively replicated by forks from neighboring origins (McCarroll and Fangman 1988; Raghuraman et al. 2001). Strikingly, it is the telomeric location—not the DNA sequence of the origins—that determines their firing efficacy (Ferguson and Fangman 1992). If an early-firing origin from elsewhere in the genome is relocated to the telomere, it will now fire late or not at all. Conversely, a telomeric origin placed on a circular plasmid will fire early and efficiently. Strikingly, this late replication of a telomeric origin is conserved in human cells (Smith and Higgs 1999). New results directly link the telomere-binding protein Rif1 to this regulation of telomeric origin firing.

Rif1 was first identified in *S. cerevisiae* as a negative telomere length regulator and helped form the basis for the protein-counting model (Hardy et al. 1992; Marcand et al. 1997; Levy and Blackburn 2004). Experiments from several different groups now show that Rif1 is an evolutionarily conserved regulator of origin firing. Deletion of *RIF1* in yeast or knockdown in mammalian cells allows the origins that were blocked in early S phase to now fire (Buonomo et al. 2009; Lian et al. 2011; Cornacchia et al. 2012; Hayano et al. 2012; Yamazaki et al. 2012; Mattarocci et al. 2014; Peace et al. 2014; Sreesankar et al. 2015). Rif1 blocks the origin firing through recruitment of protein phosphatase 1 (PP1). PP1 antagonizes the action of the DDK1 kinase, which is required for origin firing (Dave et al. 2014; Hiraga et al. 2014; Mattarocci et al. 2014). Rif1 bound at telomere repeats will thus recruit PP1 and inhibit firing of origins near the telomere. Longer telomeres would presumably have more bound Rif1, which could increase PP1 recruitment and decrease firing of telomere-proximal origins.

The replication model suggests how increasing the probability of telomeric origin firing can lead to longer telomeres in a *rif1Δ* deletion mutant. If the telomere-proximal origin does not fire due to Rif1/PP1 activity, then replication origins will have to come from the next most internal origin (Fig. 2). However, when *RIF1* is deleted, the telomere-proximal origins fire, thus decreasing the distance to the chromosome end and increasing the probability that telomerase will elongate the telomere. The replication fork model thus links the long telomeres in *RIF1* deletion mutants with their effect on origin firing.

## A feedback loop for origin activation and repression may regulate telomere length

**Figure 3.**
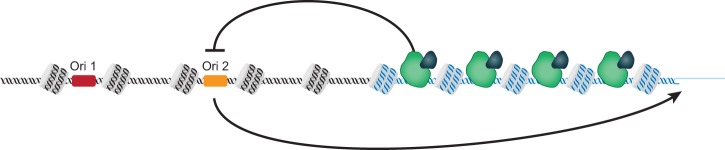
Feedback regulation of origin firing maintains telomere length homeostasis. At long telomeres, local Rif1 binding to telomere DNA (blue helix) blocks origin firing at proximal telomeres in adjacent DNA (black helix). The telomere is then replicated from the more distal Ori 1. At short telomeres, the fewer binding sites for Rif1 allows Ori 2 firing and this increases the probability of telomere extension.

Telomere length homeostasis may be established by a feedback loop between origin firing efficacy and telomere length (Fig. 3). The increased recruitment of PP1 to long telomeres decreases the probability of adjacent origin firing, and thus, over many cell cycles, long telomeres will shorten due to the end replication problem. When that telomere becomes shorter, there will be less Rif1 bound, and the telomere-proximal origin can fire again, thus increasing the probability that telomerase will arrive at the end to extend that telomere. Interestingly, when a telomere is artificially shortened, the telomere-proximal origin fires more efficiently (Bianchi and Shore 2007a), supporting a feedback mechanism between telomere length, origin activity, and telomere elongation (Fig. 3). Having outlined the general concept of the replication fork model of telomere elongation, below I examine how previous experiments that connected replication and telomere length can be interpreted in light of this model.

## Telomere elongation is linked to replication

The association of telomere length changes with DNA replication has been noted for some time, and, in fact, some elements of this model have been previously suggested in the literature. Wellinger et al. (1993) proposed that origin firing is coupled to telomere elongation and also that telomere elongation requires passage of a replication fork (Dionne and Wellinger 1998). Other groups have also linked origin firing to telomere elongation. By following elongation of an artificially shortened telomere in *S. cerevisiae* through the cell cycle, two groups found that telomere elongation by telomerase coincides exactly with telomere replication by the replisome (Marcand et al. 2000; Bianchi and Shore 2007a), and similar experiments show that this is the case in human cells as well (Hirai et al. 2012). Finally, chromatin immunoprecipitation experiments in *S. cerevisiae*; *Schizosaccharomyces pombe*, and human cells show that telomerase arrives at telomeres late in S phase. (Taggart et al. 2002; Smith et al. 2003; Bianchi and Shore 2007b; Moser et al. 2009; Hirai et al. 2012). These experiments are all consistent with the model in which telomerase arrives at the telomere with the replication fork. With a new framework for understanding the linkage of replication and telomere length, previous experimental results can be reinterpreted. For example, proteins that were proposed to “recruit” telomerase to the telomere 3′ end ssDNA overhang, such as Cdc13 and Est1 (Evans and Lundblad 1999), instead may recruit telomerase to the ssDNA created at a telomeric replication fork.

## Lagging strand DNA polymerases and Okazaki fragment processing are linked to telomere elongation

Over 30 years ago, telomere length was shown to be altered by mutations in components of lagging strand DNA synthesis (Carson and Hartwell 1985). Lagging strand replication occurs by synthesis of short stretches of DNA, called Okazaki fragments, followed by their maturation and ligation to generate a continuous DNA strand (Kurth and O'Donnell 2013). Each Okazaki fragment begins with the synthesis of a primer by the DNA polymerase α/primase complex; PCNA is then loaded and recruits DNA polymerase δ (Langston et al. 2009; O'Donnell et al. 2013). During Okazaki fragment maturation, the nascent DNA/RNA strand is processed by Fen1 and Dna2 and joined to the upstream newly synthesized DNA (Balakrishnan and Bambara 2013). After ligation, PCNA must then be unloaded from the newly synthesized DNA (Kubota et al. 2013). Strikingly, mutations in many of the components of lagging strand synthesis affect telomere length.

In *S. cerevisiae*, specific hypomorphic alleles of *POL1* (DNA polymerase α) cause excessive telomere elongation and increased telomeric ssDNA (Carson and Hartwell 1985; Adams Martin et al. 2000). Mutations in genes encoding DNA primase (Pol12), Dna2, and Fen1 that process Okazaki fragments also increase ssDNA and telomere length (Parenteau and Wellinger 1999; Grossi et al. 2004; Budd et al. 2006). Also, mutations in *PIF1*, a helicase involved in Okazaki fragment maturation (Budd et al. 2006; Bochman et al. 2010), have long telomeres (Schulz and Zakian 1994). Mutations in genes encoding in the canonical replication factor C (RFC), which loads PCNA (Adams and Holm 1996), as well as in an alternative RFC (composed of Elg1, Ctf18, and Rad24), which unloads PCNA, cause significant telomere elongation (Kanellis et al. 2003; Kubota et al. 2015). This association of lagging strand replication components, including DNA polymerase α, RFC, and Fen1, with telomere length is conserved across eukaryotes (Dahlen et al. 2003; Sampathi et al. 2009; Takashi et al. 2009; Derboven et al. 2014). The mechanism by which impairment of lagging strand synthesis might lead to telomere elongation is not clear and might seem counterintuitive. Perhaps components of the fork stabilization complex stabilize telomerase association with a stalled fork. While the mechanism is not clear, the mechanistic link between lagging strand synthesis and telomere length was further supported by Gottschling's group (Diede and Gottschling 1999). They found that DNA polymerases α and δ and DNA primase are each absolutely required for de novo telomere addition by telomerase (Diede and Gottschling 1999). Examining this link in the context of telomerase association with the fork might shed light on the mechanism of telomere length regulation.

## Telomere-specific RPA involved in telomere maintenance

The identification of a telomere-specific RPA in yeast further strengthens the link between replication and telomere length. RPA is the eukaryotic ssDNA-binding protein that binds the ssDNA behind the helicase at the fork (Fairman and Stillman 1988; Wold and Kelly 1988). The RPA complex is a trimer containing RPA70, RPA32, and RPA14 that binds to ssDNA and is required for DNA replication (Erdile et al. 1990; Brill and Stillman 1991). Work from the Lundblad laboratory (Gao et al. 2007) first suggested that Cdc13 and its two binding partners, Stn1 and Ten1, form a trimeric complex protein resembling RPA. The similarity to RPA was confirmed by the crystal structure of several domains of Stn1 and Ten1 (Gelinas et al. 2009; Sun et al. 2009), indicating that Cdc13/Stn1/Ten1 form an alternative, telomere-specific RPA complex, termed t-RPA. This telomere-specific t-RPA (also called CST) is conserved across eukaryotes. In humans, *Xenopus*, and *Arabidopsis*, the large subunit CTC1 does not share sequence identity with *CDC13*, but they do have structural similarities (Miyake et al. 2009; Surovtseva et al. 2009; Price et al. 2010). Stn1 and Ten1 are more conserved, and the crystal structure of the human STN1–TEN1 subcomplex shows structural conservation with the *S. cerevisiae* t-RPA (Bryan et al. 2013). Mutations in CTC1 cause telomere shortening in patients with human telomere syndromes (Anderson et al. 2012), and siRNA disruption of Stn1 and Ten1 increases telomere length in cultured cells (Bryan et al. 2013), highlighting the role of these proteins in mammalian length regulation.

The t-RPA interacts with polymerase α/primase (Nugent et al. 1996; Qi and Zakian 2000), as does the canonical RPA (Tsurimoto and Stillman 1989), once again linking lagging strand synthesis with telomere length regulation. A cocrystal shows binding of the Cdc13 N-terminal OB-fold domain to DNA polymerase α (Sun et al. 2011). Stn1 and Ten1 in human cells were first identified as DNA polymerase α/primase accessory proteins (Goulian et al. 1990), and biochemical reconstitution shows that the yeast t-RPA (CST) complex can stimulate DNA primase activity in vitro (Lue et al. 2014), providing functional evidence for a role in DNA replication as well as telomere length regulation.

## Conserved interaction of RPA and telomerase

If Cdc13 is a part of an alternative RPA, how do we reconcile this with its proposed role in binding of the telomere G-strand overhang and providing end protection? The fact that Cdc13 is not needed for end protection outside of S phase (Vodenicharov et al. 2010) and that chromatin immunoprecipitation (ChIP) experiments show that Cdc13 binds telomeres almost exclusively in S phase calls into question the model in which Cdc13 is constitutively bound to the telomeric G-strand overhang. Cdc13 (and the whole t-RPA complex) was proposed to associate with the single-stranded telomeric DNA at the replication fork (Gao et al. 2010), and experiments indicate that it does (Faure et al. 2010). Lundblad and colleagues (Gao et al. 2010) have proposed that t-RPA facilitates replication though telomeric DNA and prevents replication fork collapse. t-RPA binding to DNA polymerase α suggests that it is also directly linked to telomere lagging strand replication. If Cdc13 associates with single-stranded telomeric DNA just behind the fork, it would explain the finding that Cdc13 is found at telomeres in late S phase. Telomeres replicate late, and thus the single-stranded telomeric DNA would only be exposed on the lagging strand late in S phase. The association of Cdc13 with polymerase α put this alternative t-RPA squarely at the replication fork.

## Telomerase associates with t-RPA

We have known for >15 years that CDC13 binds telomerase through interaction with Est1 (Evans and Lundblad 1999). The recent cryo-electron microscopy (cryo-EM) structure of the *Tetrahymena* telomerase holoenzyme also directly links telomerase to t-RPA. The structure shows that two distinct t-RPA complexes are bound with TERT in the telomerase holoenzyme (Jiang et al. 2015). These studies provide compelling evidence that telomerase may associate with the replication fork though its interaction with t-RPA, and thus this complex is one candidate for a factor that might mediate telomerase association with the replication fork, although other models for recruiting telomerase to the fork should also be considered.

As discussed above, in ciliates, telomerase travels with replication bands that represent synchronous replication forks. The telomere-binding protein TBPα/TBPβ from *Oxytricha nova* (Fang and Cech 1995) and *Euplotes* (Skopp et al. 1996) also travels with the fork, further suggesting that TBPα/TBPβ may be part of a t-RPA that associates with telomerase.

## Conclusions

The protein-counting model explains some of the experimental data on the negative regulation of telomere length but does not account for the role of origin firing or lagging strand synthesis in regulating length. When a model is drawn many times in articles and reviews, it influences how scientists interpret their experiments. Many aspects of this replication fork model still need to be tested. However, even if some aspects are not borne out, examining telomere regulation from a new angle may still inspire new interpretations of both past and future experiments. This model, I hope, will be refined or replaced as we learn more about how the cell maintains telomeres in an exquisite balance between lengthening and shortening.
